# Diosgenin Induces Apoptosis in HepG2 Cells through Generation of Reactive Oxygen Species and Mitochondrial Pathway

**DOI:** 10.1155/2012/981675

**Published:** 2012-06-06

**Authors:** Dae Sung Kim, Byoung Kook Jeon, Young Eun Lee, Won Hong Woo, Yeun Ja Mun

**Affiliations:** ^1^Department of Herbal Resources, Professional Graduate School of Oriental Medicine, Wonkwang University, Republic of Korea; ^2^Department of Food and Nutrition, College of Environmental Resources, Wonkwang University, Republic of Korea; ^3^Department of Anatomy, College of Oriental Medicine, Wonkwang University, Republic of Korea

## Abstract

Diosgenin, a naturally occurring steroid saponin found abundantly in legumes and yams, is a precursor of various synthetic steroidal drugs. Diosgenin is studied for the mechanism of its action in apoptotic pathway in human hepatocellular carcinoma cells. Based on DAPI staining, diosgenin-treated cells manifested nuclear shrinkage, condensation, and fragmentation. Treatment of HepG2 cells with 40 *μ*M diosgenin resulted in activation of the caspase-3, -8, -9 and cleavage of poly-ADP-ribose polymerase (PARP) and the release of cytochrome *c.* In the upstream, diosgenin increased the expression of Bax, decreased the expression of Bid and Bcl-2, and augmented the Bax/Bcl-2 ratio. Diosgenin-induced, dose-dependent induction of apoptosis was accompanied by sustained phosphorylation of JNK, p38 MAPK and apoptosis signal-regulating kinase (ASK)-1, as well as generation of the ROS. NAC administration, a scavenger of ROS, reversed diosgene-induced cell death. These results suggest that diosgenin-induced apoptosis in HepG2 cells through Bcl-2 protein family-mediated mitochndria/caspase-3-dependent pathway. Also, diosgenin strongly generated ROS and this oxidative stress might induce apoptosis through activation of ASK1, which are critical upstream signals for JNK/p38 MAPK activation in HepG2 cancer cells.

## 1. Introduction

Diosgenin is a steroidal saponin, which is found in a variety of plants including fenugreek (*Trigonella foenum graecum*), roots of wild yam (*Dioscorea villosa*), *Solanum incaunm,* and *Solanum xanthocarpum* [1]. It has been reported to have various effects, such as a hypocholesterolemic action in rat, or an antioxidant activity in HIV patients with dementia [2, 3]. Diosgenin has been shown to exert anticancer effects against a wide variety of tumor cells, including breast cancer, colorectal cancer, osteosarcoma, and leukemia [4–7]. The antitumor effects of diosgenin have been demonstrated to be mediated through activation of p53, immune-modulation, cell cycle arrest, modulation of caspase-3 activity, and induction of TRAIL death receptor DR5 [8–10]. A recent study has shown that diosgenin inhibited proliferation and induced apoptosis in HepG2 cells by inhibiting signal transducer and activator of transcription (STAT3) signaling pathway [11].

Apoptosis is a programmed cell death process that controls normal development and homeostasis in organisms. The loss of apoptotic control contributes to the survival of tumor cells, and the enhancement of cancer cell apoptosis is one approach of controlling cancer by anticancer agents [12]. At the biochemical level, apoptosis is mediated by the activation of a class of cysteine proteases called caspases. In mammalian cells, caspase activation mainly occurs either through death receptor activation or mitochondrial membrane permeabilization [13]. The mitochondrial pathway of apoptosis is regulated principally by the Bcl-2 protein family. In response to apoptotic signals, Bax, a proapoptotic member of the Bcl-2 family, is redistributed from the cytosol to the mitochondria. Conversely, overexpression of Bcl-2 protects apoptosis. Therefore, the ratio of expression of the proapoptotic Bax protein and the antiapoptotic Bcl-2 protein ultimately determines cell death or survival in this mitochondrial death pathway [14, 15].

One of the well-known intracellular signaling pathways for apoptosis is the kinase cascade, which has been identified as a transducing pathway of apoptotic signals initiated by outside stimuli, mitogen-activated protein (MAP) kinases, and their upstream kinases such as MAP kinase kinases [16]. Many stimuli such as anticancer drugs, irradiation, TNF-*α*, and chemopreventive agents prompt cells to produce ROS [17, 18]. It has been shown that ROS induces a number of events including mitogen-activated protein kinases (MAPKs) signal transduction pathways in mediating apoptosis [19, 20].

Apoptosis signal-regulating kinase (ASK)-1 is a member of the ROS-sensitive MAP kinase kinases and it acts as a redox sensor of cells [21]. ASK 1 is activated in response to TNF-*α*, Fas, and oxidative stress. Overexpression of inactive ASK1 can inhibit TNF-*α* or Fas-ligand-induced cell death [22]. On the other hand, constitutively active ASK1 overexpression has been shown to cause apoptosis through mitochondrial-dependent caspase activation [23]. Thus, ASK1 appears to be a key player in the MAPK (p38 MAPK/JNK) control of cell death and cell survival.

Diosgenin has been shown to target multiple pathways of tumorigenesis, including proliferation, apoptosis, angiogenesis, invasion, and tumor-induced immunosuppression in various tumor cells and *in vivo* cancer models [1]. However, no reports exist in the literature elaborating the effect of diosgenin on ROS-ASK1-MAPK signaling cascade in HepG2 cells. In this study, we investigated the involvement of ASK1 in the apoptotic process of HepG2 cells treated with a chemopreventive agent, diosgenin. Here, we demonstrated that diosgenin strongly generated ROS and this oxidative stress induced apoptosis through activation of ASK1, which are critical upstream signals for p38 MAPK/JNK activation in HepG2 cancer cells.

## 2. Material and Methods

### 2.1. Cell Culture and Drug Treatment

Human hepatoma cell line (HepG2) was cultured in RPMI (Gibco) supplemented with 10% fetal bovine serum (Gibco). The cells were cultured at 37°C in a humidified chamber with 95% air and 5% CO_2_. All experiments were performed in plastic tissue culture flasks (Falcon). HepG2 cells were seeded on 24 well plates or 100 mm culture dishes. After plating, cells were allowed to adhere overnight and were then treated with chemical. Diosgenin was purchased from Sigma and stored at −20°C. Diosgenin stock solutions were made in ethanol (100%) and diluted in medium prior to use.

### 2.2. Determination of Cell Viability (MTT Assay)

Cell viability was determined by the MTT [3-(4,5-dimethylthiazol-2yl)-2,5-diphenyltetrazolium bromide] assay. The cells were seeded in 24-well plates at a density of 4 × 10^4^ cells/well and treated with Diosgenin at various concentration (0–40 *μ*M) for 24 h and 48 h. After the exposure period, media were removed. Thereafter, the medium was changed and incubated with MTT (0.1 mg/mL) for 3 h. The viable cell number per dish is directly proportional to the production of formazan, which was solubilized in isopropanol, and measured spectrophotometrically at 570 nm.

### 2.3. Apoptosis Assays

Fluorescence-associated cell sorting (FACS) analysis was performed to discriminate between intact and apoptotic cells. Staining for FITC-labeled annexin V binding to membrane phosphatidylserine and propidium iodide (PI) binding for cellular DNA was performed according to the protocol provided by the manufacturer (Boehringer Mannheim). Briefly, cells (1 × 10^6^ cells) were suspended in buffer containing FITC-conjugated annexin V and PI at appropriate concentrations. The samples were analyzed by FACS Vantage using Cell Quest Software (Beckton Dickinson) and 20,000 events from each sample were acquired to ensure adequate data.

### 2.4. DAPI Staining

Cells (1 × 10^5^) were plated onto 18 mm^2^ coverslips in flasks and cultured with complete medium. After they were treated with diosgenin, the cells were fixed with 4% formaldehyde for 20 min at room temperature and were then washed with PBS. Cold methanol was added for another 20 min at room temperature followed by washes with PBS by three times. The membrane permeable fluorescent dye DAPI (2 *μ*g/mL), which binds to chromatin of cells, was added to the fixed cells, and the cells were examined by an inverted Olympus IX70 microscope (Japan). Apoptotic cells were identified by condensation and fragmentation of nuclei. For each experiment, nuclei from 10 random fields of each coverslip were examined at ×200 magnification.

### 2.5. Western Blot Analysis

After the indicated diosgenin treatment, the medium was removed, and the cells were rinsed with PBS twice. After the addition of 0.6 mL of cold RIPA buffer (10 mM Tris pH 7.5, 100 mM NaCl, 1 mM EDTA, 0.5% Na-deoxycholate, 0.1% SDS, 1% Triton X 100) and protease inhibitors, cells were scraped followed at 4°C. Cell lysate was then subjected to a centrifugation of 14,000 × g for 15 min at 4°C. Resultant protein samples were separated by an SDS-PAGE gel and transferred onto a polyvinylidine difloride membrane (PVDF, Millipore) membrane. Membrane was stained by ponceu to confirm uniform transfer of all samples and then incubated in blocking solution (PBS with 0.05% tween 20 and 5% non fat dry-milk) for 1 h at room temperature. The antibodies used in this study, caspase-3, caspase-8, caspase-9, Bcl-2, Bax, Bid and cytochrome *c* were obtained from Santa Cruz Biotechnology Inc (Santa Cruz, CA), and p38, JNK, phospho-p38, and phosphor-JNK were purchased from Upstate Cell Signaling. The membrane was reacted firstly with desired primary antibodies for 1 h at room temperature. Membrane was then incubated with appropriate horseradish peroxidase-conjugated secondary antibody (Zymed) for 1 h, washed with PBST, and developed using the ECL kit.

### 2.6. ROS Assay

Intracellular generation of ROS was measured with carboxy-H_2_DCFDA (Invitrogen), which is a cell-permeable and nonfluorescent dye when loaded onto the cells. This compound is oxidized by ROS to fluorescent carboxydichlorofluorescein (DCF) inside the cells. Briefly, the cells seeded in 6-well plates (2 × 10^5^ cells/well) and treated with or without diosgenin were incubated with 5 *μ*M carboxy-H_2_DCFDA for 15 min at 37°C. Then the cells were washed with phosphate buffered saline (PBS) twice, trypsinized, and resuspended in OptiMem I medium. The fluorescence resulting from the rate of oxidation of the dye in the cells was measured using a FACS with an excitation wavelength of 480 nm and an emission wavelength of 530 nm. The generation of ROS in HepG2 cells was also verified by fluorescence microscopy (Nicon, Japen). Cells grown to confluence were treated with or without diosgenin in the presence of 5 *μ*M carboxy-H_2_DCFDA for the indicated time and resuspended in fresh OptiMem I medium after washing. During fluorescence imaging, the illumination level was reduced to minimal level to prevent photosensitization of the fluorescent probe.

### 2.7. Statistical Analysis

All experiments were performed in triplicates and the results were expressed as mean ± S.D. Statistical significances were analyzed by one-way analysis of variance (ANOVA) with Duncan test. *P* value ≤ 0.05 was considered statistically significant (STATSTICA 2.0, USA).

## 3. Results and Discussion

### 3.1. Cytotoxic Effect of Diosgenin on HepG2 Cells

**Figure 1:**
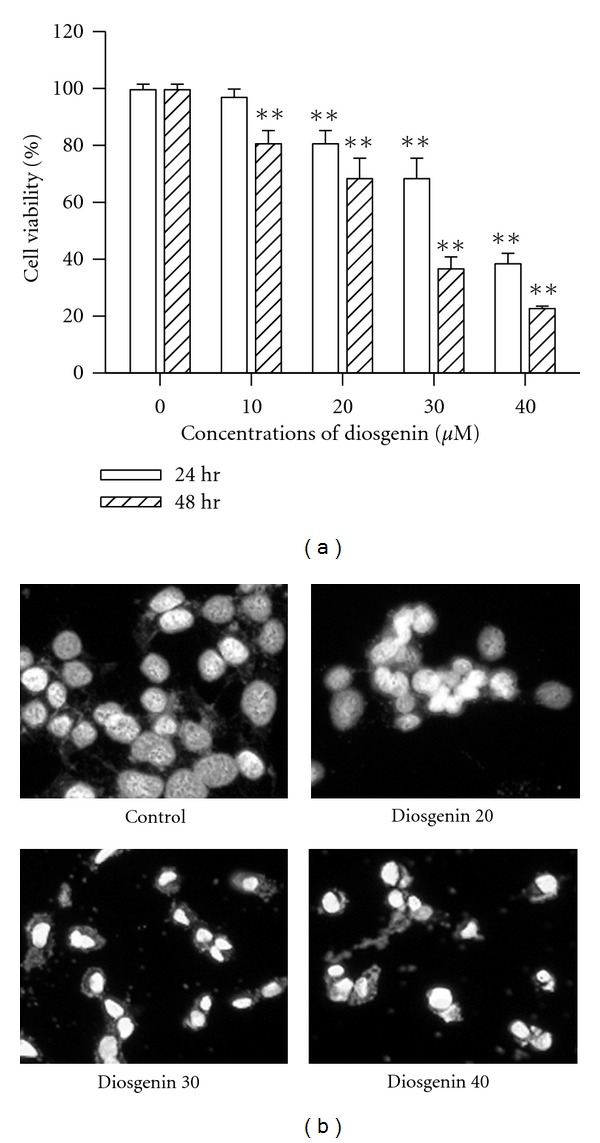
Cytotoxic Effect of diosgenin in HepG2 cells. (a) Cells were treated with diosgenin by dose-dependent manner for 24 and 48 h. The ratios of cell viability were measured by MTT assay. Data are presented as mean ± SD of six replicates from three independent experiments. **P < 0.01 compared to control. (b) Nuclear alterations were observed by DAPI staining and fluorescence microscopy (×100). After cells were treated with diosgenin (0–40 *μ*M) for 48 h, marked morphological changes of cell apoptosis such as condensation of chromatin and nuclear fragmentations were found clearly using DAPI staining.

**Figure 2:**
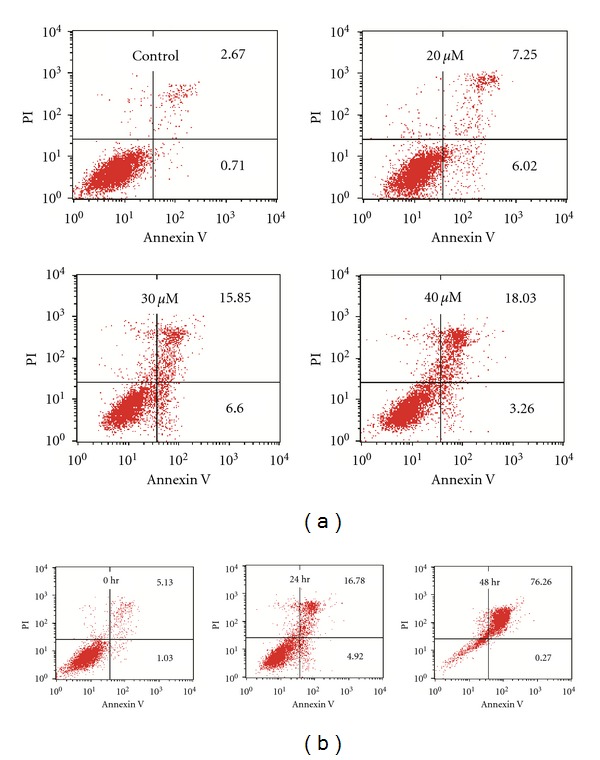
FACS analyses of Annexin V and PI staining. HepG2 cells was treated with diosgenin (0–40 *μ*M) for 24 h (a) and 40 *μ*M for 0, 24, 48 h (b). Lower right quadrant, early apoptosis cells, that is, Annexin V-FITC-positive/PI-negative cells; upper right quadrant, necrosis or late-apoptotic cells, that is, Annexin V-FITC-positive/PI-positive cells.

A previous study has shown that diosgenin inhibited proliferation and induced apoptosis in HepG2 cells [11]. To confirm whether diosgenin influences the viability of hepatoma cells, HepG2 cells were challenged with diosgenin (0–40 *μ*M). Cytotoxicity is measured by MTT assay following a brief during exposure. Diosgenin markedly induced cell death in HepG2 cells in a dose- and time-dependent manner as compared with vehicle controls (Figure 1). Apoptosis is initially characterized by morphological features, such as chromatin condensation, nuclear fragmentation, and membrane blebbing [24]. In the current study, morphological changes of cell apoptosis such as condensation of chromatin and nuclear fragmentation were clearly observed by DAPI staining after 24 h of diosgenin (Figure 1). Cell death was also assessed with flow cytometry after double staining with annexin V and PI. We challenged the cells with increasing doses of diosgenin at 24 h of treatment. According to Figure 2(a), the combined early and late apoptotic cells (Annexin V positive) were elevated in a dose-dependent fashion. Consistent with the progression of apoptosis, late apoptotic cells become dominant at later time, because we observed gradual diminution of early apoptotic cells and increment of the late apoptotic cells after 48 h (Figure 2(b)). These findings demonstrate that diosgenin induced the apoptosis of HepG2 cells in both dose- and time-dependent manners.

### 3.2. Effect of Diosgenin on Activation of Caspases and Bcl-2 Family

**Figure 3:**
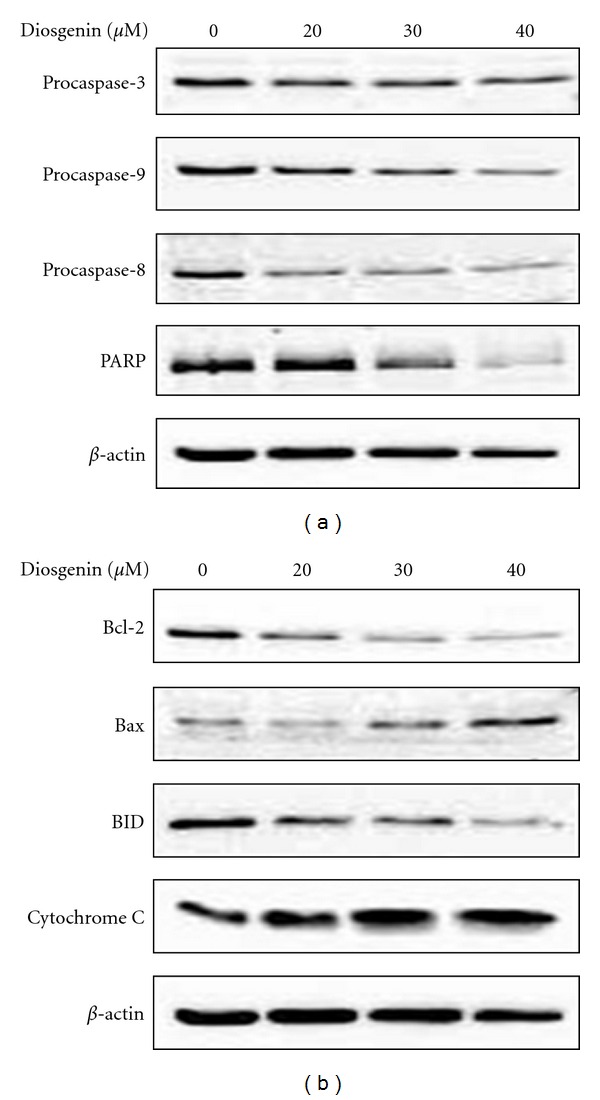
Effects of diosgenin on caspases (a), Bcl-2 family proteins, and cytochrome *c* (b). Cells were incubated without or with 20 *μ*M, 30 *μ*M, and 40 *μ*M of diosgenin for 24 h. Total cell lysates were analyzed by immunoblotting with antibody against caspase-3 (pro and cleavage), procaspase-8, procaspase-9, PARP, Bcl-2, Bax, Bid, and cytochrom *c*.

Caspase activation is generally considered to be a key hallmark of apoptosis. Mitochondria are involved in a variety of key events leading to apoptosis, as releasing of caspase activators, the production of reactive oxygen species (ROS), and participation in regulation of both pro- and anti-apoptotic bcl-2 family proteins [8]. In the next series of experiment, we assessed the effect of diosgenin on the cascade of caspases that are crucial initiators and effectors in various cell death pathways. As shown in Figure 3(a), diosgenin treatment activated caspase-3, caspase-8 and caspase-9 (as shown by decreased procaspase-9 and -8 levels) followed by subsequent PARP cleavage.

Bcl-2 family proteins are crucial for apoptosis commitment, mainly via the control of the mitochondrial pathway which is frequently triggered in response to chemotherapeutic agents [25]. Elevated levels of Bcl-2 in tumor cells may contribute to chemoresistance by stabilizing the mitochondrial membrane against apoptotic insult. Bax and Bak are the critical effectors of apoptosis acting downstream of the both the prosurvival and BH3-only members [26]. The BH3-only proteins (Bid, Bim, Puma, Noxa) are pro-apoptotic and act as sensors of specific types of cellular stress [27]. Thus, Bcl-2 family proteins may be good therapeutic targets.

To investigate the cellular mechanism underlying diosgenin-induced apoptosis in HepG2 cells, we analyzed the expression of apoptosis-regulated genes; including pro-survial Bcl-2, pore-forming Bax and pro-apoptotic Bid proteins. As illustrated in Figure 3(b), the expression level of Bcl-2 was gradually down-regulated as the diosgenin concentration increased. In addition, diosgenin significantly induced the activation of Bax and Bid. Bid functions to receive death signals in the cytosol from upstream events and is cleaved to truncated Bid (tBid; 15). Upon translocation of Bid to mitochondria, it induces the release of cytochrome *c*. From subsequent experiments, we observed a higher expression of cytochrome *c*. Cytochrome *c* release is not a specific sign of apoptosis, which also occurs during necrotic cell death [28]. However, our results also revealed the increased levels of caspases and Bax and the decreased levels of Bcl-2 and Bid. These results indicate that diosgenin leads to a shift from antiapoptosis to proapoptosis by altering the function of the proteins in the Bcl-2 family, which results in the release of cytochrome *c* from mitochondria.

### 3.3. Diosgenin-Induced Apoptosis by the Generation of Reactive Oxygen Species (ROS)

**Figure 4:**
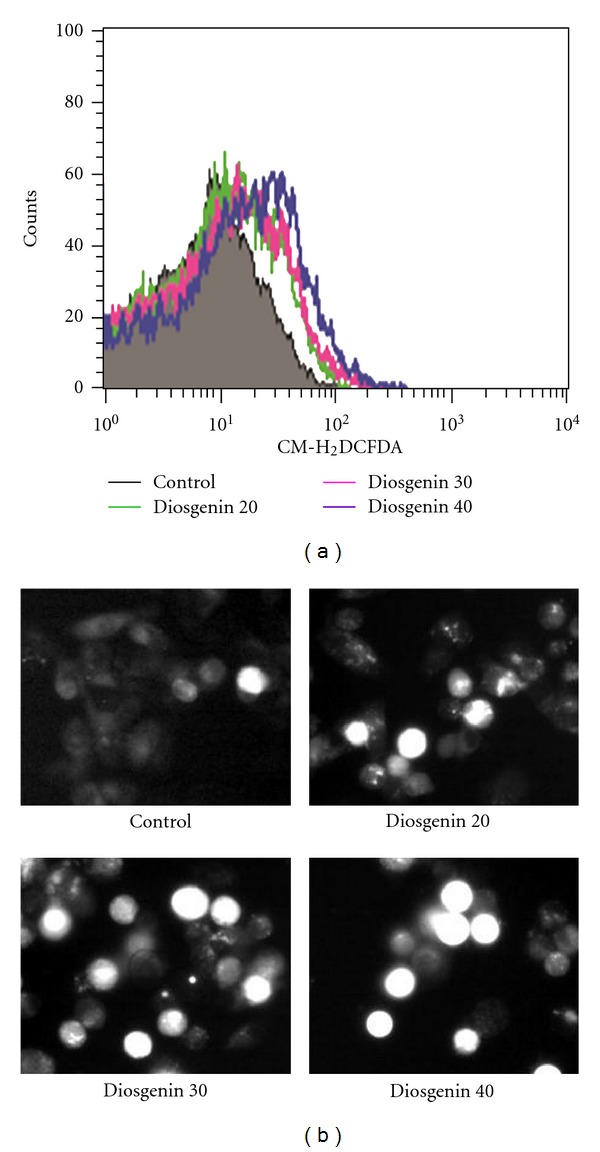
Diosgenin generated ROS in HepG2 cells. (a) Diosgenin generated ROS in HepG2 cells. Cells were treated with different concentrations of diosgenin (20–40 *μ*M) plus 40 *μ*M of DCFH-DA for 24 h, and ROS productions were determined by FACS analysis. (b) For observation of intracellular ROS by fluorescence microscope, cells were treated with diosgenin (20–40 *μ*M) and then incubated with DCFH-DA.

**Figure 5:**
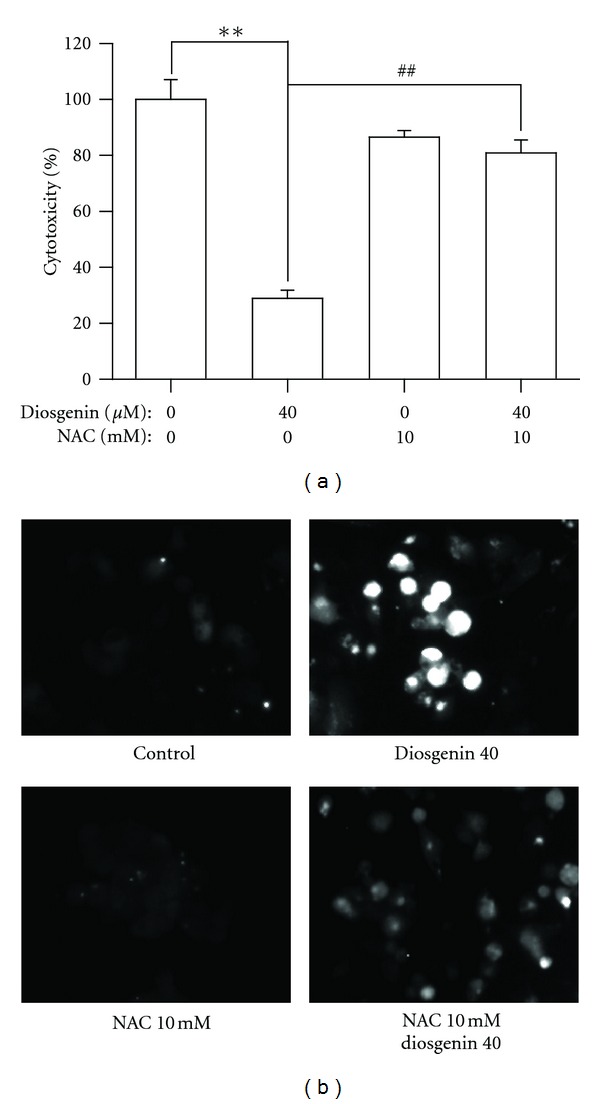
Diosgenin-generated ROS induced apoptosis in HepG2 cells. (a) The cells were pretreated with/without NAC (10 mM) at least 2 hr before the treatment of 40 *μ*M diosgenin. After 24 h, quantitative assessment of the percentage of cell viability was determined by MTT assay. **P < 0.01 compared to control, ^##^ *P* < 0.01 compared to diosgenin 40 *μ*M-treated group. (b) Also cells were pretreated with/without NAC (10 mM) at least 2 h before the treatment of diosgenin. ROS production was confirmed by fluorescence microscope.

As reactive oxygen species (ROS) generation is an important role in apoptosis, we investigated the ability of diosgenin to generate ROS. Cells were exposed to diosgenin (0–40 *μ*M) for 24 hr and analyzed for the present of ROS by flow cytometry The generation of ROS by diosgenin was increased in dose-dependent manner (Figure 4(a)). We also confirmed intracellular ROS production by fluorescence microscope after staining with carboxy-H_2_DCFDA, ROS were generated by treatment of diosgenin (Figure 4(b)). To examine whether diosgenin-generated ROS induce apoptosis in HepG2 cells, we measured cell death after treatment of diosgenin only or with NAC. NAC is a potent antioxidant that can inhibit oxidative stress by directly scavenging ROS and replenishing GSH [29]. If ROS production mediates diosgenin-induced cell death, we expect that NAC should have the ability to inhibit diosgenin-induced cell death. As shown in Figure 5(a), diosgenin (40 *μ*M) increased cell death, whereas removing diosgenin-generated ROS by NAC led to decreasing cell death. Also, the decrement of intracellular ROS by treating NAC was observed after DCFH-DA staining (Figure 5(b)). These results indicate that diosgenin induced cell death of HepG2 cells by the generation of ROS.

### 3.4. Diosgenin-Activated MAPKs and ASK1

**Figure 6:**
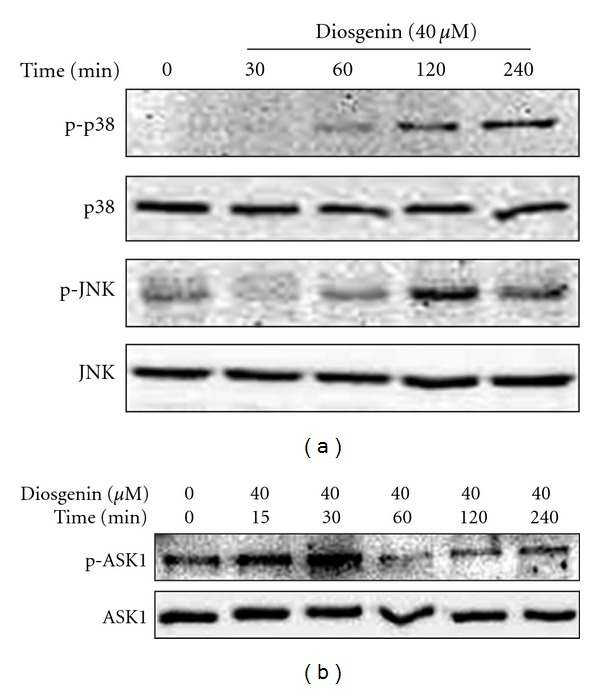
Effects of diosgenin on MAPK (a) and ASK1 (b). Cells were treated with varying concentrations of diosgenin (20–40 *μ*M) for 24 h. Total cell lysates were analyzed by immunoblotting with antibody against phospho-p38, p38, phospho-JNK, JNK, phosphoASK1, and ASK1.

Intracellular MAPKs are the major oxidative stress-sensitive signal transduction pathways [16]. The major enzymes belonging to MAPKs are the extracellular signal regulating kinase 1/2(ERK1/2 or p44/42 MAPK), c-Jun N-terminal kinase (JNK), and p38 MAPK. JNK and p38 are stress-activated MAP kinases that are preferentially activated by cytotoxic stress, such as X-ray/UV irradiation, heat/osmotic shock, and oxidative/nitrosative stress [19, 30]. To identify the signaling pathways involved in diosgenin-induced cell death, HepG2 cells were treated with 40 *μ*M diosgenin, and activation of MAPKs was determined by Western blotting. As illustrated in Figure 6(a), p38 MAPK and JNK activation in HepG2 cells was induced by diosgenin.

 Next, we measured the phosphorylation levels of ASK1 for determining ASK1 activation. Our results showed that ASK1 phosphorylation was increased at 60 min, and sustained to 240 min by diosgenin (Figure 6(b)). ASK1 is an upstream kinase of JNK and p38 MAPK [31]. JNK and p38 MAPK are activated through ASK1 in response to various extracelluar stimuli [21, 32]. The binding of ASK1 to TNF receptor-associated factor or death domain-associated protein stimulates ASK1 function, whereas the ASK1 inhibitory proteins such as thioredoxin downregulate proapoptotic activity of ASK1 [33, 34]. Recent studies have suggested that ROS-mediated ASK1 activation is involved in a variety of disorders, such as inflammation [13, 14], neurodegeneration [15, 16], and cardiac hypertrophy and remodeling [18, 19]. It has already been reported that EGCG and berberine executed apoptotic cell death via an ASK1 and JNK/p38 cascade, which is induced by NAC-sensitive intracellular oxidation or ROS [35–37]. In this study, diosgenin activated JNK, p38 MAPK and ASK1 as well as ROS generation. Therefore, further studies on the mechanisms of regulation of ASK1 activity and the development of ASK1-targeting drugs may contribute to the treatment of various diseases caused by oxidative stress.

## 4. Conclusion

In conclusion, the present data showed that diosgenin induced apoptosis in HepG2 cells through Bcl-2 protein family- (Bcl-2, Bax, and Bid)-mediated mitochondria/caspase-3-dependent pathway. Also, diosgenin strongly generated ROS and this oxidative stress might induce apoptosis through activation of ASK1, which are critical upstream signals for JNK/p38 MAPK activation in HepG2 cancer cells.
